# Volume 33 Index

**Published:** 2011

## Author Index

### A

Amedee, A.M.

  Alcohol’s Role in HIV Transmission and Disease Progression

  Vol. 33, No. 3, Pages 203–218

Anthenelli, R.M.

  Focus On: Comorbid Mental Health Disorders

  Vol. 33, Nos. 1 and 2, Pages 109–117

### B

Bagby, G.J.

  Alcohol’s Role in HIV Transmission and Disease Progression

  Vol. 33, No. 3, Pages 203–218

Barve, S.

  Focus on the Liver: Alcohol Use, Highly Active Antiretroviral Therapy, and Liver Disease in HIV-Infected Patients

  Vol. 33, No. 3, Pages 229–236

Boyle, M.G.

  An E-Health Solution for People With Alcohol Problems

  Vol. 33, No. 4, Pages 327–337

Braithwaite, R.S.

  Integrating HIV/AIDS and Alcohol Research

  Vol. 33, No. 3, Pages 167–178

  Influence of Alcohol Consumption on Adherence to and Toxicity of Antiretroviral Therapy and Survival

  Vol. 33, No. 3, Pages 280–287

Bryant, K.J.

  Integrating HIV/AIDS and Alcohol Research

  Vol. 33, No. 3, Pages 167–178

  Influence of Alcohol Consumption on Adherence to and Toxicity of Antiretroviral Therapy and Survival

  Vol. 33, No. 3, Pages 280–287

### C

Caetano, R.

  Ethnicity and Health Disparities in Alcohol Research

  Vol. 33, Nos. 1 and 2, Pages 152–160

Chartier, K.

  Ethnicity and Health Disparities in Alcohol Research

  Vol. 33, Nos. 1 and 2, Pages 152–160

Chi, F.

  Integrating Care for People With Co-Occurring Alcohol and Other Drug, Medical, and Mental Health Conditions

  Vol. 33, No. 4, Pages 338–349

Cunningham, J.A.

  The Use of Emerging Technologies in Alcohol Treatment

  Vol. 33, No. 4, Pages 320–326

### E

Eaton, J.W.

  Focus on the Liver: Alcohol Use, Highly Active Antiretroviral Therapy, and Liver Disease in HIV-Infected Patients

  Vol. 33, No. 3, Pages 229–236

### F

Fell, J.C.

  Preventing Alcohol-Related Problems Through Health Policy Research

  Vol. 33, Nos. 1 and 2, Pages 18–28

Fiellin, D.

  HIV/AIDS, Comorbidity, and Alcohol: Can We Make a Difference?

  Vol. 33, No. 3, Pages 258–266

Freiberg, M.S.

  Focus on the Heart: Alcohol Consumption, HIV Infection, and Cardiovascular Disease

  Vol. 33, No. 3, Pages 237–246

### G

Gobejishvili, L.

  Focus on the Liver: Alcohol Use, Highly Active Antiretroviral Therapy, and Liver Disease in HIV-Infected Patients

  Vol. 33, No. 3, Pages 229–236

Guidot, D.M.

  Focus on the Lung

  Vol. 33, No. 3, Pages 219–228

Gustafson, D.H.

  An E-Health Solution for People With Alcohol Problems

  Vol. 33, No. 4, Pages 327–337

### H

Happel, K.I.

  Focus On: Alcohol and the Immune System

  Vol. 33, Nos. 1 and 2, Pages 97–108

  Alcohol’s Role in HIV Transmission and Disease Progression

  Vol. 33, No. 3, Pages 203–218

Harris, R.A.

  Alcohol’s Effects on Brain and Behavior

  Vol. 33, Nos. 1 and 2, Pages 127–143

Hewitt, B.G.

  NIAAA: Advancing Alcohol Research for 40 Years

  Vol. 33, Nos. 1 and 2, Pages 5–17

  Fetal Alcohol Spectrum Disorders: From Research to Policy

  Vol. 33, Nos. 1 and 2, Pages 118–126

Hiller-Sturmhöfel, S.

  Treating Alcoholism As a Chronic Disease: Approaches to Long-Term Continuing Care

  Vol. 33, No. 4, Pages 356–370

Hingson, R.W.

  Focus on: College Drinking and Related Problems

  Magnitude and Prevention of College Drinking and Related Problems

  Vol. 33, Nos. 1 and 2, Pages 45–54

Hinman, A.

  Integrating Care for People With Co-Occurring Alcohol and Other Drug, Medical, and Mental Health Conditions

  Vol. 33, No. 4, Pages 338–349

Horgan, C.M.

  Health Services and Financing of Treatment

  Vol. 33, No. 4, Pages 389–394

Huebner, R.B.

  Advances in Alcoholism Treatment

  Vol. 33, No. 4, Pages 295–299

### I

Isham, A.

  An E-Health Solution for People With Alcohol Problems

  Vol. 33, No. 4, Pages 327–337

### J

Johnson, K.

  An E-Health Solution for People With Alcohol Problems

  Vol. 33, No. 4, Pages 327–337

Joshi-Barve, S.

  Focus on the Liver: Alcohol Use, Highly Active Antiretroviral Therapy, and Liver Disease in HIV-Infected Patients

  Vol. 33, No. 3, Pages 229–236

Justice, A.

  HIV/AIDS, Comorbidity, and Alcohol: Can We Make a Difference?

  Vol. 33, No. 3, Pages 258–266

### K

Kalichman, S.C.

  Social and Structural HIV Prevention in Alcohol-Serving Establishments: Review of International Interventions Across Populations

  Vol. 33, No. 3, Pages 184–194

Kapoor, R.

  Focus on the Liver: Alcohol Use, Highly Active Antiretroviral Therapy, and Liver Disease in HIV-Infected Patients

  Vol. 33, No. 3, Pages 229–236

Kelly, J.F.

  The Role of Mutual-Help Groups in Extending the Framework of Treatment

  Vol. 33, No. 4, Pages 350–355

Kolls, J.K.

  Focus On: Alcohol and the Immune System

  Vol. 33, Nos. 1 and 2, Pages 97–108

Koob, G.F.

  Focus On: Neuroscience and Treatment The Potential of Neuroscience to Inform Treatment

  Vol. 33, Nos. 1 and 2, Pages 144–151

Kraemer, K.L.

  Focus on the Heart: Alcohol Consumption, HIV Infection, and Cardiovascular Disease

  Vol. 33, No. 3, Pages 237–246

Kypros, K.

  The Use of Emerging Technologies in Alcohol Treatment

  Vol. 33, No. 4, Pages 320–326

### L

Levy, M.

  An E-Health Solution for People With Alcohol Problems

  Vol. 33, No. 4, Pages 327–337

### M

Mandrekar, P.

  Focus On: Alcohol and the Liver

  Vol. 33, Nos. 1 and 2, Pages 87–96

Marlatt, G.A.

  Behavioral Therapy Across the Spectrum

  Vol. 33, No. 4, Pages 313–319

Mayer, K.H.

  Biomedical Approaches to HIV Prevention

  Vol. 33, No. 3, Pages 195–202

McCambridge, J.

  The Use of Emerging Technologies in Alcohol Treatment

  Vol. 33, No. 4, Pages 320–326

McClain, C.J.

  Focus on the Liver: Alcohol Use, Highly Active Antiretroviral Therapy, and Liver Disease in HIV-Infected Patients

  Vol. 33, No. 3, Pages 229–236

McKay, J.R.

  Treating Alcoholism As a Chronic Disease: Approaches to Long-Term Continuing Care

  Vol. 33, No. 4, Pages 356–370

McTavish, F.

  An E-Health Solution for People With Alcohol Problems

  Vol. 33, No. 4, Pages 327–337

Meyers, R.J.

  The Community Reinforcement Approach: An Update of the Evidence

  Vol. 33, No. 4, Pages 380–388

Mimiaga, M.J.

  Biomedical Approaches to HIV Prevention

  Vol. 33, No. 3, Pages 195–202

Mochly-Rosen, D.

  Focus On: The Cardiovascular System—What Did We Learn From the French (Paradox)?

  Vol. 33, Nos. 1 and 2, Pages 76–86

Moghe, A.

  Focus on the Liver: Alcohol Use, Highly Active Antiretroviral Therapy, and Liver Disease in HIV-Infected Patients

  Vol. 33, No. 3, Pages 229–236

Molina, P.E.

  Focus On: Alcohol and the Immune System

  Vol. 33, Nos. 1 and 2, Pages 97–108

### N

Nelson, S.

  Focus On: Alcohol and the Immune System

  Vol. 33, Nos. 1 and 2, Pages 97–108

  Integrating HIV/AIDS and Alcohol Research

  Vol. 33, No. 3, Pages 167–178

  Alcohol’s Role in HIV Transmission and Disease Progression

  Vol. 33, No. 3, Pages 203–218

### O

O’Connor, P.G.

  Medications for Unhealthy Alcohol Use: Across the Spectrum

  Vol. 33, No. 4, Pages 300–312

O’Malley, S.S.

  Medications for Unhealthy Alcohol Use: Across the Spectrum

  Vol. 33, No. 4, Pages 300–312

### P

Pandrea, I.

  Alcohol’s Role in HIV Transmission and Disease Progression

  Vol. 33, No. 3, Pages 203–218

Pfefferbaum, A.

  Alcohol’s Effects on Brain and Behavior

  Vol. 33, Nos. 1 and 2, Pages 127–143

  Focus on the Brain: HIV Infection and Alcoholism: Comorbidity Effects on Brain Structure and Function

  Vol. 33, No. 3, Pages 247–257

### Q

Quintero, D.

  Focus on the Lung

  Vol. 33, No. 3, Pages 219–228

### R

Ramirez, J.A.

  Focus on the Liver: Alcohol Use, Highly Active Antiretroviral Therapy, and Liver Disease in HIV-Infected Patients

  Vol. 33, No. 3, Pages 229–236

Richards, S.

  An E-Health Solution for People With Alcohol Problems

  Vol. 33, No. 4, Pages 327–337

Roach, D.

  Integrating HIV/AIDS and Alcohol Research

  Vol. 33, No. 3, Pages 167–178

Robinson, W.

  HIV Risk and the Alcohol Environment: Advancing an Ecological Epidemiology for HIV/AIDS

  Vol. 33, No. 3, Pages 179–183

Roozen, H.G.

  The Community Reinforcement Approach: An Update of the Evidence

  Vol. 33, No. 4, Pages 380–388

Rosenbloom, M.J.

  Focus on the Brain: HIV Infection and Alcoholism: Comorbidity Effects on Brain Structure and Function

  Vol. 33, No. 3, Pages 247–257

### S

Samet, J.H.

  Interventions Targeting HIV-Infected Risky Drinkers: Drops in the Bottle

  Vol. 33, No. 3, Pages 267–279

Schubert, C.

  An E-Health Solution for People With Alcohol Problems

  Vol. 33, No. 4, Pages 327–387

Scribner, R.

  HIV Risk and the Alcohol Environment: Advancing an Ecological Epidemiology for HIV/AIDS

  Vol. 33, No. 3, Pages 179–183

Shaw, B.R.

  An E-Health Solution for People With Alcohol Problems

  Vol. 33, No. 4, Pages 327–337

Simonson, N.

  HIV Risk and the Alcohol Environment: Advancing an Ecological Epidemiology for HIV/AIDS

  Vol. 33, No. 3, Pages 179–183

Simpson, C.A.

  The Recovery Spectrum: From Self-Change to Seeking Treatment

  Vol. 33, No. 4, Pages 371–379

Skeer, M.

  Biomedical Approaches to HIV Prevention

  Vol. 33, No. 3, Pages 195–202

Smith, J.E.

  The Community Reinforcement Approach: An Update of the Evidence

  Vol. 33, No. 4, Pages 380–388

Sterling, S.

  Integrating Care for People With Co-Occurring Alcohol and Other Drug, Medical, and Mental Health Conditions

  Vol. 33, No. 4, Pages 338–349

Stewart, M.T.

  Health Services and Financing of Treatment

  Vol. 33, No. 4, Pages 389–394

Sullivan, E.V.

  Alcohol’s Effects on Brain and Behavior

  Vol. 33, Nos. 1 and 2, Pages 127–143

  Focus on the Brain: HIV Infection and Alcoholism: Comorbidity Effects on Brain Structure and Function

  Vol. 33, No. 3, Pages 247–257

Sullivan, L.

  HIV/AIDS, Comorbidity, and Alcohol: Can We Make a Difference?

  Vol. 33, No. 3, Pages 258–266

Szabo, G.

  Focus On: Alcohol and the Liver

  Vol. 33, Nos. 1 and 2, Pages 87–96

### T

Theall, K.P.

  HIV Risk and the Alcohol Environment: Advancing an Ecological Epidemiology for HIV/AIDS

  Vol. 33, No. 3, Pages 179–183

Thoman, J.D.

  Fetal Alcohol Spectrum Disorders: From Research to Policy

  Vol. 33, Nos. 1 and 2, Pages 118–126

Tucker, J.A.

  The Recovery Spectrum: From Self-Change to Seeking Treatment

  Vol. 33, No. 4, Pages 371–379

### V

Voas, R.B.

  Preventing Alcohol-Related Problems Through Health Policy Research

  Vol. 33, Nos. 1 and 2, Pages 18–28

### W

Walley, A.Y.

  Interventions Targeting HIV-Infected Risky Drinkers: Drops in the Bottle

  Vol. 33, No. 3, Pages 267–279

Warren, K.R.

  NIAAA: Advancing Alcohol Research for 40 Years

  Vol. 33, Nos. 1 and 2, Pages 5–17

  Fetal Alcohol Spectrum Disorders: From Research to Policy

  Vol. 33, Nos. 1 and 2, Pages 118–126

Willenbring, M.L.

  The Past and Future of Research on Treatment of Alcohol Dependence

  Vol. 33, Nos. 1 and 2, Pages 55–63

Windle, M.

  Reducing Underage and Young Adult Drinking: How to Address Critical Drinking Problems During This Developmental Period

  Vol. 33, Nos. 1 and 2, Pages 29–44

Witkiewitz, K.

  Behavioral Therapy Across the Spectrum

  Vol. 33, No. 4, Pages 313–319

Wolfgang Kantor, L.

  Advances in Alcoholism Treatment

  Vol. 33, No. 4, Pages 295–299

### Y

Yeterian, J.D.

  The Role of Mutual-Help Groups in Extending the Framework of Treatment

  Vol. 33, No. 4, Pages 350–355

### Z

Zakhari, S.

  Focus On: The Cardiovascular System—What Did We Learn From the French (Paradox)?

  Vol. 33, Nos. 1 and 2, Pages 76–86

Zhang, P.

  Focus On: Alcohol and the Immune System

  Vol. 33, Nos. 1 and 2, Pages 97–108

Zucker, R.A.

  Reducing Underage and Young Adult

  Drinking: How to Address Critical Drinking Problems During This Developmental Period

  Vol. 33, Nos. 1 and 2, Pages 29–44

## Index of Articles

### A

Advances in Alcoholism Treatment

  R.B. Huebner with L. Wolfgang Kantor

  Vol. 33, No. 4, Pages 295–299

Alcohol’s Effects on Brain and Behavior

  E.V. Sullivan, R.A. Harris, and A. Pfefferbaum

  Vol. 33, Nos. 1 and 2, Pages 127–143

Alcohol’s Role in HIV Transmission and Disease Progression

  I. Pandrea, K.I. Happel, A.M. Amedee, G.J. Bagby, and S. Nelson

  Vol. 33, No. 3, Pages 203–218

An E-Health Solution for People With Alcohol Problems

  D.H. Gustafson, M.G. Boyle, B.R. Shaw, A. Isham, F. McTavish, S. Richards, C. Schubert, M. Levy, and K. Johnson

  Vol. 33, No. 4, Pages 327–337

### B

Behavioral Therapy Across the Spectrum

  K. Witkiewitz and G.A. Marlatt

  Vol. 33, No. 4, Pages 313–319

Biomedical Approaches to HIV Prevention

  K.H. Mayer, M. Skeer, and M.J. Mimiaga

  Vol. 33, No. 3, Pages 195–202

### C

Ethnicity and Health Disparities in Alcohol Research

  K. Chartier and R. Caetano

  Vol. 33, Nos. 1 and 2, Pages 152–160

### F

Fetal Alcohol Spectrum Disorders: From Research to Policy

  J.D. Thomas, K.R. Warren, and B.G. Hewitt

Vol. 33, Nos. 1 and 2, Pages 118–126

Focus On: Alcohol and the Immune System

  P.E. Molina, K.I. Happel, P. Zhang, J.K. Kolls, and S. Nelson

  Vol. 33, Nos. 1 and 2, Pages 97–108

Focus On: Alcohol and the Liver

  G. Szabo and P. Mandrekar

  Vol. 33, Nos. 1 and 2, Pages 87–96

Focus on: College Drinking and Related Problems

  Magnitude and Prevention of College Drinking and Related Problems

  R.W. Hingson

  Vol. 33, Nos. 1 and 2, Pages 45–54

Focus On: Comorbid Mental Health Disorders

  R.M. Anthenelli

  Vol. 33, Nos. 1 and 2, Pages 109–117

Focus On: Neuroscience and Treatment

  The Potential of Neuroscience to Inform

  Treatment

  G.F. Koob

  Vol. 33, Nos. 1 and 2, Pages 144–151

Focus on the Brain: HIV Infection and Alcoholism: Comorbidity Effects on Brain Structure and Function

  M.J. Rosenbloom, E.V. Sullivan, and A. Pfefferbaum

  Vol. 33, No. 3, Pages 247–257

Focus On: The Cardiovascular System—What Did We Learn From the French (Paradox)?

  D. Mochly-Rosen and S. Zakhari

  Vol. 33, Nos. 1 and 2, Pages 76–86

Focus on the Heart: Alcohol Consumption, HIV

  Infection, and Cardiovascular Disease

  M.S. Freiberg and K.L. Kraemer

  Vol. 33, No. 3, Pages 237–246

Focus on the Liver: Alcohol Use, Highly Active

  Antiretroviral Therapy, and Liver Disease in HIV-Infected Patients

  S. Barve, R. Kapoor, A. Moghe, J.A. Ramirez, J.W. Eaton, L. Gobejishvili, S. Joshi-Barve, and C.J. McClain

  Vol. 33, No. 3, Pages 229–236

Focus on the Lung

  D. Quintero and D.M. Guidot

  Vol. 33, No. 3, Pages 219–228

### G

Genetic Research: Who Is At Risk for Alcoholism?

  T. Foroud, H.J. Edenberg, and J.C. Crabbe

  Vol. 33, Nos. 1 and 2, Pages 64–75

### H

Health Services and Financing of Treatment

  M.T. Stewart and C.M. Horgan

  Vol. 33, No. 4, Pages 389–394

HIV/AIDS, Comorbidity, and Alcohol: Can We Make a Difference?

  A. Justice, L. Sullivan, and D. Fiellin

  Vol. 33, No. 3, Pages 258–266

HIV Risk and the Alcohol Environment: Advancing an Ecological Epidemiology for HIV/AIDS

  R. Scribner, K.P. Theall, N. Simonsen, and W. Robinson

  Vol. 33, No. 3, Pages 179–183

### I

Influence of Alcohol Consumption on Adherence to and Toxicity of Antiretroviral Therapy and Survival

  R.S. Braithwaite and K.J. Bryant

  Vol. 33, No. 3, Pages 280–287

Integrating Care for People With Co-Occurring

  Alcohol and Other Drug, Medical, and Mental Health Conditions

  S. Sterling, F. Chi, and A. Hinman

  Vol. 33, No. 4, Pages 338–349

Integrating HIV/AIDS and Alcohol Research

  K.J. Bryant, S. Nelson, R.S. Braithwaite, and D. Roach

  Vol. 33, No. 3, Pages 167–178

Interventions Targeting HIV-Infected Risky

  Drinkers: Drops in the Bottle

  J.H. Samet and A.Y. Walley

  Vol. 33, No. 3, Pages 267–279

### M

Medications for Unhealthy Alcohol Use: Across the Spectrum

  S.S. O’Malley and P.G. O’Connor

  Vol. 33, No. 4, Pages 300–312

### N

NIAAA: Advancing Alcohol Research for 40 Years

  K.R. Warren and B.G. Hewitt

  Vol. 33, Nos. 1 and 2, Pages 5–17

### P

Preventing Alcohol-Related Problems Through

  Health Policy Research

  R.B. Voas and J.C. Fell

  Vol. 33, Nos. 1 and 2, Pages 18–28

### R

Reducing Underage and Young Adult Drinking: How to Address Critical Drinking Problems During This Developmental Period

  M. Windle and R.A. Zucker

  Vol. 33, Nos. 1 and 2, Pages 29–44

### S

Social and Structural HIV Prevention in Alcohol-

  Serving Establishments: Review of International Interventions Across Populations

  S.C. Kalichman

  Vol. 33, No. 3, Pages 184–194

### T

The Community Reinforcement Approach: An Update of the Evidence

  R.J. Meyers, H.G. Roozen, and J.E. Smith

  Vol. 33, No. 4, Pages 380–388

The Past and Future of Research on Treatment of Alcohol Dependence

  M.L. Willenbring

  Vol. 33, Nos. 1 and 2, Pages 55–63

The Recovery Spectrum: From Self-Change to Seeking Treatment

  J.A. Tucker and C.A. Simpson

  Vol. 33, No. 4, Pages 371–379

The Role of Mutual-Help Groups in Extending the Framework of Treatment

  J. F. Kelly and J.D. Yeterian

  Vol. 33, No. 4, Pages 350–355

The Use of Emerging Technologies in Alcohol Treatment

  J.A. Cunningham, K. Kypri, and J. McCambridge

  Vol. 33, No. 4, Pages 320–326

Treating Alcoholism As a Chronic Disease: Approaches to Long-Term Continuing Care

  J. R. McKay and S. Hiller-Sturmhöfel

  Vol. 33, No. 4, Pages 356–370

